# An Overview of Dengue Knowledge, Attitudes, and Practices (KAPs) Among the General Public in Sri Lanka: A Review and Meta-Analysis of Questionnaire-Based Surveys from 2000–2023

**DOI:** 10.3390/tropicalmed10070189

**Published:** 2025-07-06

**Authors:** Nilmini Chandrasena, Dileepa Ediriweera, Deshaka Jayakody, Nayana Gunathilaka, Ranjan Premaratna

**Affiliations:** 1 Department of Parasitology, Faculty of Medicine, University of Kelaniya, Ragama 11010, Sri Lanka; djayak.mem2403@pg.kln.ac.lk (D.J.); n.gunathilaka@kln.ac.lk (N.G.); 2 Health Data Science Unit, Faculty of Medicine, University of Kelaniya, Ragama 11010, Sri Lanka; dileepa@kln.ac.lk; 3 Department of Medicine, Faculty of Medicine, University of Kelaniya, Ragama 11010, Sri Lanka; ranjanp64@kln.ac.lk

**Keywords:** dengue, knowledge, attitudes, practices, Sri Lanka

## Abstract

The objective was to conduct a review and meta-analysis of questionnaire-based surveys of dengue knowledge, attitudes, perceptions, and practices (KAP)s among the general public in Sri Lanka as no prior island-wide survey existed. The electronic database PubMed and other bibliography were searched for literature on dengue questionnaire-based KAP surveys in Sri Lanka from 2000–2023. Data pertaining to the three domains were extracted from sixteen eligible articles, pooled, and analyzed separately using random effect models. Meta-analyses of the three domains were performed using R version 3.6.3. The population surveyed (8955) was <0.045% of the total Sri Lankan population. The publication frequency increased over time and surveys were distributed in Colombo and suburbs 43.7% (7/16), Kandy 25% (4/16,) Gampaha 12.5% (2/16), and 6.3% (1/16) one each in Kurunegala, Matara, Batticaloa, and Jaffna. Knowledge on dengue transmission, vector breeding, and fever as a symptom was >80%, while on vector species, preferred feeding times, recurrence of dengue it was > 55% and on warning signs of severity it was 25%. Attitudes towards community participation in dengue prevention activities and knowledge of dengue risk factors (avoidance of aspirin and dark colored drinks) were poor, while practice of control measures (removal of water collecting receptacles, roof-gutter management) lacked regularity.

## 1. Introduction

Dengue fever, a mosquito-borne viral infection, stands as the most widespread and rapidly expanding arboviral infection globally [1]. The illness presents as a spectrum of disease manifestations and is categorized into dengue fever (DF) with or without warning signs and severe dengue which includes Dengue Hemorrhagic Fever (DHF)/Dengue Shock Syndrome (DSS) and severe organ impairment as defined by the World Health Organization (WHO) 2009 [2]. The global incidence of dengue has markedly increased over the past two decades [3]. Currently, over half the global population is at risk in 129 countries, with an estimated 10,000 deaths and 100 million symptomatic infections per year [4]. Thus, dengue is an ever-present threat to human health in tropical and subtropical regions of the world where the mosquito vectors *Aedes aegypti* and *A. albopictus* thrive. Approximately 70% of the global burden of dengue is estimated to be in Asia [3].

Sri Lanka is a tropical island nation (population 22 million) situated in the Indian Ocean comprised of nine administrative provinces and 26 districts. Among the Asian countries with a high dengue burden, Sri Lanka ranks amongst the world’s 30 most endemic countries [5]. Following the first serological confirmationof dengue in Sri Lanka in 1962, multiple island-wide outbreaks of DF with cases of severe dengue (DHF, DSS) and deaths occurred [6,7,8,9]. The magnitude of dengue epidemics increased from early 2000 onwards, with large epidemics documented in years 2002, 2004 and 2017 [8]. In 2005, the National Dengue Control Unit (NDCU) of the Ministry of Health was established to curb the escalating burden of dengue cases [8]. The NDCU implemented dengue prevention programs at district and national level. Pivotal to the sustainability of dengue control is involvement of the community. Thus, improving public awareness, attitudes, and practices on all aspects of dengue has been the focus of social mobilization programs conducted by the NDCU [8]. Activities include declaration of dengue mosquito control week/s, production and dissemination of dengue information, education, and communication (IEC) material to the public aimed at enhancing or creating awareness on preventive strategies and to promote early health-seeking behavior [8]. The communication for behavioral impact (COMBI) approach was implemented to encourage specified behaviors (removal of breeding sites and early medical attention for fever) to target groups (housewives, school principals and teachers, dengue prevention committees and dengue patients) with key messages comprising weekly inspections and clearance of vector breeding sites in home, school, and work environments, seeking treatment for fevers of over two days from a qualified medical doctor and avoidance of anti-inflammatory drugs [8]. These promotional activities mostly happen periodically in anticipation of or during an outbreak of dengue. The impact of these societal interventions has been studied in Sri Lanka via questionnaire-based surveys on KAPs targeting specified communities or subpopulations who were perceived as high-risk groups (school children) or were important in implementation of control strategies (housewives, school teachers, and youth organizations). However, an island-wide survey or a review of dengue KAPs has not reported to date. Thus, the objective was to conduct a review of the literature on questionnaire-based dengue KAP surveys in Sri Lanka with meta-analysis of the three domains.

## 2. Materials and Methods

### 2.1. Search Strategy

A search of the PubMed database was conducted on the 28 December 2023 to identify relevant literature from year 2000 onwards using relevant Medical Subject Headings (MeSH) terms “Dengue/epidemiology”, “Dengue/prevention & control”, “Sri Lanka”, and variations of “Knowledge, Attitudes, and Practices.” Additional search techniques were employed to broaden the search. These techniques included searching within titles and abstracts, incorporating alternative keywords and phrases, and exploring related articles and citations. The search was expanded to include terms such as “survey”, “questionnaire”, “prevention”, and “treatment” within titles and abstracts. We also used wildcards and truncation to capture variations of terms, ensuring that our search was not restricted by specific wording. The titles and abstracts of the search output were screened independently by two investigators (Nilmini Chandrasena and Nayana Gunathilake) for relevance and eligibility based on inclusion criteria. In the event of controversy, a third investigator (Ranjan Premaratna) was consulted.

### 2.2. Inclusion Criteria

The inclusion criteria comprised of cross-sectional studies with quantitative data on dengue-related KAPs were conducted in Sri Lanka and utilized questionnaire-based methodologies. The titles and abstracts of the articles were screened by three authors and those not meeting the inclusion criteria were excluded.

### 2.3. Data Extraction for Analysis

Two investigators (Deshaka Jayakody and Nilmini Chandrasena) performed data extraction in which DJ extracted data into a pre-formed data extraction template while NC cross-checked the extracted data. The data extracted were the reported outcomes of survey questions addressing any of the KAP domains per study. Outcomes reported as percentages were converted to numbers where necessary. Survey items across studies were categorized under the three domains. The items under the knowledge domain included knowledge on viral etiology, transmission, vector bionomics, symptoms of infection, warning signs of severity, re-infection events, preventive, and management measures (hydration, avoidance of aspirin and dark colored drinks or food). Items under the attitudes domain included perceptions of severity, treatability, preventability, treatment-seeking behaviors, support for premise inspection, and participation in community clean-up activities. The items under the practices domain included practice of individual protection measures (mosquito nets, repellants) and activities pertaining to prevention of mosquito breeding (environmental clean-up, covering water-holding containers, clearance of roof gutters). A table was created to organize data, accounting for variability in study tools and surveyed items.

### 2.4. Meta-Analysis of Data in the Three Domains

There were differences in the number of questions in the surveys. We, therefore, considered each question as a separate outcome and conducted the analysis. To assess the collective outcome of each question within KAP surveys, we conducted a separate meta-analysis. Heterogeneity between studies was evaluated using the I^2^ statistic and the Cochran Q test. A random-effects meta-analysis of proportions was conducted to calculate pooled estimates, accounting for heterogeneity [10]. Inverse variance weighting and the DerSimonian–Laird method were used to pool the estimates [11]. Statistical analyses were performed (Dileepa Ediriweera) using R version 3.6.3. The “meta” package was used for meta-analyses of proportions. The “dmetar” package was used to identify outliers and influential studies.

## 3. Results

**Figure 1:**
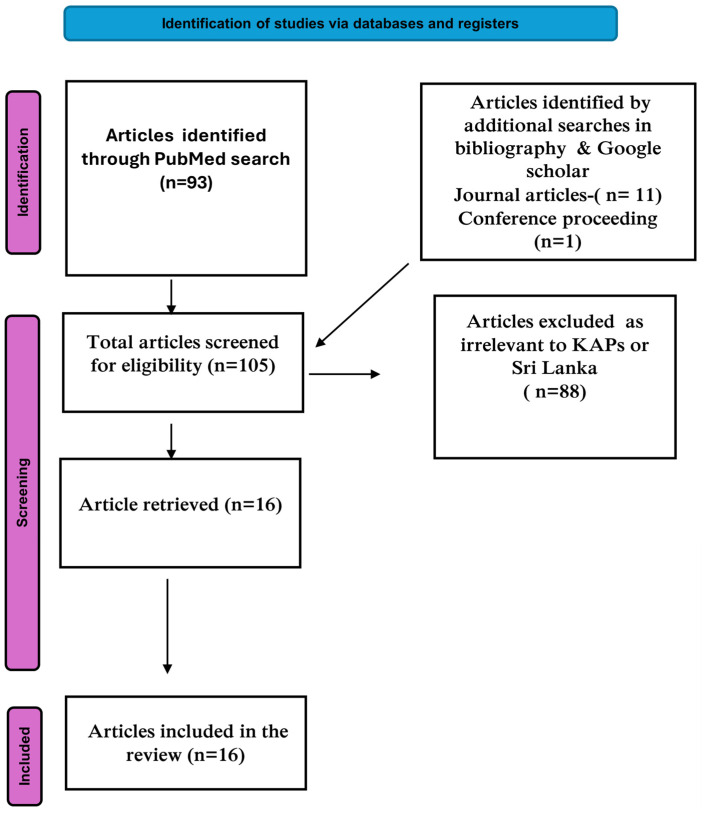
Flowchart of papers included in the review.

A total of 93 articles were obtained from the PubMed search using MeSH terms. Additionally, 11 more articles were found through bibliography and Google Scholar searches, adding up to a total of 105 articles. Of these, 88 were excluded, as these were determined to be irrelevant to KAP studies and/or Sri Lanka. Ultimately, a total of 16 articles were included in the review (Figure 1).

Questionnaire-based KAP surveys on dengue were published from 2012 onwards in Sri Lanka with only five surveys being reported during the first six years of scrutiny (2012–2017) compared to eleven studies being documented over the subsequent six years (2018–2023). The majority of the studies were conducted in urban and semi-urban areas in the districts of Colombo and suburbs 43.7% (7/16,) Kandy 25% (4/16), and Gampaha 12.5% (2/16), while the districts of Kurunegala, Matara, Batticaloa, and Jaffna reported a single study each. The population screened for dengue KAPs in Sri Lanka was a minority (0.045% n = 8955). Specified populations were mostly targeted such as community members residing in high-endemic areas (seven studies, n = 4667), high-risk populations such as school children (two studies, n = 2331), undergraduates (single study, n = 384), hospital-based studies on patients receiving indoor care for dengue (two studies, n = 332) outpatients (single study, n = 500), parents of children hospitalized for fever (single study, n = 86) and subpopulations involved in implementing control, such as housewives (single study, n = 400), school teachers (single study, n = 105), urban youths (single study, n = 150), (Table 1).

### 3.1. Study Tool Validity and Reliability

The study tools utilized were validated and pre-tested in three studies [12,14,22], only pre-tested in five [13,16,23,28,29], and only validated in four [18,19,24,30] studies. Two studies reported the reliability of the study tool by calculating the Cronbach’s α coefficients with scores ranging from 0.83–0.98 [12,24]. The questionnaires were self-administered (n = 8) or interviewer-administered (n = 7) while one was administered online via a social media-network.

### 3.2. Knowledge Domain

Meta-analysis of the knowledge domain indicates that the Sri Lankan population in urban and suburban areas had good (≥80%) knowledge on transmission aspects of dengue [13,16,17,19,21,22,25,26], vector bionomics which included breeding preferences [14,16,17,18,20,23,25,26] the day-biting nature of vectors [15,16,17,18,19,22,26], and viral etiology [14,26]. The knowledge of mosquito vector species was 55% [13,16,17,19,20,21,22,25,26], but the vector adaptations to breed in brackish and polluted water were limited [13,14]. Knowledge of dengue as a febrile infection was good (>80%) [13,14,15,16,17,19,20,21,22,26]. However, knowledge of other symptoms (abdominal pain, rash,) or severity indicators such as low-urine output and bleeding manifestations was low (25%) (Table 2).

Knowledge of the potential for recurrence of dengue due to multiple viral serotypes [13,17,18,19,26] and association of outbreaks with rainy season was >60% [14,15,16,22]. Less than half (41%) were aware that there was no specific medicine to cure dengue [15,18,20]. The awareness of dengue risks was surprisingly low (27.9%) in some high-endemic residential environments [25,27]. Factors affecting the knowledge domain were level of education [12,13,17,18,19,22], family income [12,17,22], and past dengue experience [22,25]. Media (television and radio broad casts) were preferred sources of information [13,16,19], while newspapers, schools, and health personnel also contributed [13,16,19] (Table 2).

### 3.3. Attitudes Domain

Attitudes towards perceived dengue severity with potential for fatality [15,17,18,19], susceptibility regardless of age [13], treatability [13,17], preventability [19], early treatment-seeking behavior from qualified medical practitioners [13,14,17,20,21,22,24], support for premise inspection and fogging [15,22,26] were positive (>75%) (Table 3). Attitudes towards maintaining hydration during illness [13,14,19,21,22] were average (>55%). Only a minority had positive attitudes towards Ayurveda (5%), while beliefs on papaya leaf extracts to cure dengue had more acceptability (32% were strongly positive and 22% were positive) [17,20]. Negative attitudes were reported with regard to the perceived onus of dengue control activities, with 73.6% perceiving it as the government’s responsibility [12,22], 35% perceiving it as individual’s responsibility [14], and 58% perceiving it a shared responsibility between the state and individuals [17,22] (Table 3). Factors having a positive impact on attitudes domain were high family income, male gender, past dengue experience, employment status, and health awareness programs tailored for specific demographic groups [12,22,23,24].

### 3.4. Practices Domain

The practice of mosquito source reduction measures such as clearance of water collecting receptacles outdoors [15,16,18,21,24], covering water tanks, wells and containers [12,15], and cleaning and scrubbing outdoor water-holding containers [15,16,17,19,21,22,24,26] was good (>70%), but management of indoor breeding sites was barely adequate (<60%) [13,22,24] (Table 4). The management of roof gutters was less than adequate (44.5%) [17,24]. Personal protection methods were practiced by <55%; these include the use of mosquito nets 45.7% [15,17], mosquito coils 43.8% [15,17,19,26], repellants 19.3% [17,19] insecticides 27% [17,19,22] and protective clothing 54.7% [15] (Table 4).

Except for physical rest implemented by 71.1% [13,14], other practices related to home-based care of dengue patients were barely adequate (<60%); paracetamol for fever management was 57.3% [13,14,19], avoidance of non-steroidal anti-inflammatory drugs (NSAID)s was 32.9% [16,17], and avoidance of dark colored drinks was 37.1% [13,17,18] (Table 4). Factors influencing positive practices were age (25–30 years), dengue knowledge, positive attitudes, family income, unemployment (practices were better among the unemployed), and past dengue experience [12,22] (Table 4).

## 4. Discussion

This review provides an overview of dengue KAP studies conducted in Sri Lanka from 2000–2023. Although the number of studies included in this review was low, this review is the first to report an overall assessment of dengue KAPs in Sri Lanka. On comparison with dengue KAP reviews in the region, the number of studies in Sri Lanka was similar in range to that of Philippines but low compared to Malaysia and high compared to Thailand [28,29,30]. The population screened was just a minority.

Meta-analysis of the knowledge domain indicates that the Sri Lankan population in urban and suburban areas had good (≥80%) knowledge on transmission dynamics and viral etiology and febrile nature of dengue infection. (see Table 2). These knowledge rates were much higher than those reported for the Southeast Asian region [31]. Awareness of basic illness features of dengue in Sri Lanka (flue-like illness) was consistent with studies in the region (Philippines, Laos) [28,32]. The low knowledge (25%) on severity indicators such as rash, abdominal pain, and bleeding manifestations was similar to a report in Laos where most (>70%) knew that dengue caused fever but only 18.2% knew of rash, and 3% of bleeding tendencies [32].

The knowledge gaps on warning signs could lead to delayed hospitalization and fatalities. Good public knowledge on dengue transmission may be attributed to the consistent efforts of the Ministry of Health and NDCU to educate the general public on dengue prevention during or prior to outbreaks. The high literacy rates (males, 93.63% and females, 91.3%) particularly in the urban and suburban settings perhaps play a role in the assimilation of the core health messages. These findings are consistent with studies reported in Vietnam, Malaysia, and Tamil Nadu [33,34,35]. In contrast, in the Philippines, the level of education was inversely associated with dengue knowledge [28].

Health education is an important component of dengue control as a correlation with increased mortality and low dengue knowledge has been documented [30]. Deficiencies noted on home-management aspects such as avoidance of NSAIDS and dark colored drinks and foods such as coke, coffee, and chocolate require more focus in awareness programs. Therefore, developing a national communication strategy that is consistent and timely to build long-term national awareness is suggested. Positive attitudes towards dengue severity perceptions and management in Sri Lanka were similar to those reported in Vietnam, Nepal, and Yemen [33,36,37]. Seeking early medical assistance (Western medicine) was comparable to Philippines, Vietnam, and Tamil Nadu [28,33,35]. In contrast, a study in Malaysia reported that only 50.8% would seek medical assistance if a child with dengue was restless or lethargic [29].

Despite the low faith in Ayurvedic treatment, positive beliefs on papaya leaf extracts were reported. Complimentary substances such as papaya leaf extracts with reported platelet-increasing properties were highly used in Malaysia and the Philippines [29,38]. The negative attitude towards dengue control responsibilities in Sri Lanka was similarly reported in the Philippines, Vietnam, and Tamil Nadu [28,33,35]. However, attitudes towards supporting premise inspection and fogging were positive (>75%) in Sri Lanka. Conduction of awareness programs in schools and other educational institutes on a regular basis may be an option for instilling a sense of community responsibility and thereby improve attitudes among the younger generations [23]. The value of school-based health education programs for dengue control has been widely reported in the literature [39,40].

The practice of source reduction measures was mostly limited to house and garden premises and lacked regularity [13,16]. Public areas such as roads, sidewalks, and neighboring premises were not attended due to time constraints and or fear of disrupting public relations. Attention to water collecting receptacles indoors (58.3%) and management of roof gutters (44.5%) was less than adequate. Regional disparities were noted in application of preventive practices with certain regions focusing more on individual protection methods, or source reduction while some practiced both [12,13,16,19,25]. Public satisfaction was reportedly low regarding the state control efforts [20,21]. Instead of penalizing the public for not clearing vector breeding sites in their properties, a more community-friendly approach by the local authorities is suggested, particularly in clean-up of state-owned public places. Regular removal of solid waste (weekly or biweekly) by the municipality is mandatory to maintain environments free of clutter. The COMBI approach needs to be reinforced and strengthened to motivate the public to change their risk-behaviors.

## 5. Limitations

The study outcomes may not be representative of dengue KAPs in the general population of Sri Lanka as most surveys targeted specific populations. The variations of the study tools (questionnaires) may have affected the outcome of the review while the comprehensiveness of the literature search may have been limited by the selectivity of MESH terms used in the search. The responses in the domains of attitudes and practices may have been influenced by social desirable bias. Temporal changes on knowledge and behaviors patterns were not analyzed as targeted populations differed.

## 6. Conclusions

Knowledge on dengue transmission and basic illness features among the general public of Sri Lanka was good, but there were notable gaps in the knowledge domain on dengue severity indicators and risk factors. Negative attitudes towards dengue control responsibilities, deficiencies in home-based care practices, and irregularity in removal of breeding sites (source reduction practices) were the gaps identified in attitudes and practices domains. Geographic disparities in KAP surveys suggest less focus on peripheral districts that are now reporting dengue cases. These geographic gaps need to be addressed by policymakers and researchers in the future. Concerted efforts are required to bridge the identified gaps. The need for a national communication strategy on dengue that is consistent and timely to build long-term national awareness, a dedicated communication expert, community-capacity building programs for provision of technical skills in maintenance of environments is recommended. Reinforcing the COMBI approach to motivate the public to change their risk-behaviors and the integration of dengue control with other vector-borne disease control programs are suggested for cost-efficacy.

## Figures and Tables

**Table 1:** Publications included in the review on dengue knowledge, attitudes, and practices surveys in Sri Lanka.

Author	Article Title	Year	Region of Sri Lanka	Sample Size	Age Group	Study Population	Main Outcomes of the Study
Chanyasanha et al. [12]	Factors influencing preventive behaviors for dengue infection among housewives in Colombo, Sri Lanka	2015	Colombo municipality	400	20+	Housewives	69.2% had dengue knowledge 91.5% positive attitudes 58.5% preventive knowledge
Disanayaka et al. [13]	Knowledge, attitudes, and practices (KAP) about dengue prevention among residents in Ratmalana medical officer of health area	2017	Attidiya North	312	18+	Community members (Ratmalana MOH area)	>90% had dengue knowledge >90% positive attitudes to early medical attention and source reduction 65% participation in environmental clean ups
Gayathri et al. [14]	Knowledge, attitudes, and practices towards dengue among undergraduate students at a University in Sri Lanka	2021	University of Peradeniya	384	20–30	Undergraduate students	40–60% in health-related faculties had satisfactory knowledge <20% in non-health-related faculties had satisfactory knowledge Overall, 60.9% had fair-satisfactory practice scores
Gunadhasam et al. [15]	Knowledge, attitudes, and practices on dengue prevention among secondary school students in university community project area	2021	Batticaloa District	137	14–16	Secondary school students	68.6% had good knowledge 67.9% had good practices 44.5% had poor attitudes
Gunasekara et al. [16]	Knowledge, attitudes, and practices regarding dengue fever in a suburban community in Sri Lanka	2012	Colombo district	349	<20–55+	Community members (Boralesgamuwa MOH area)	58% had satisfactory knowledge score (>70) 37% had satisfactory attitudes (score > 75) 85% had good practices; (score > 70)
Jayalath et al. [17]	Knowledge and attitudes regarding dengue fever among the outdoor patients of the teaching hospital Peradeniya, Sri Lanka	2018	Teaching hospital Peradeniya	500	20–70	Outpatients at Teaching Hospital Peradeniya	46.5% had >average knowledge
Jayawickreme et al. [18]	A study on knowledge, attitudes, and practices regarding dengue fever, its prevention and management among dengue patients presenting to a tertiary care hospital in Sri Lanka	2021	Colombo	132	13+	Dengue patients at SJH	62% had dengue prevention awareness 51% had dengue management awareness
Kumanan et al. [19]	A study on knowledge, attitudes, and practices regarding dengue among hospitalized patients from Northern Sri Lanka	2018	Jaffna	200	12+	Dengue patients at Teaching Hospital Jaffna	>90%–had early health-seeking behavior, Prevention, mostly avoidance of mosquito bites, with low focus on source reduction
Nazeer et al. [20]	Awareness of dengue fever among the urban youth in Colombo and its Suburbs, Sri Lanka in November 2014	2015	Colombo and suburbs	150	16–25	Urban youth	48% knew dengue specific features Awareness on substances to avoid during illness; 66% NSAIDS 16% unspecified food items 32% believed in papaya leaf extract therapy
Pavithra et al. [21]	Awareness, attitudes, and preventive measures practiced towards dengue fever by the teachers of three schools in Colombo District	2015	Colombo District	105	20–60	Teachers in the Colombo District	20% had good knowledge 68% had fair knowledge 12% poor knowledge 65% regarded prevention a self-responsibility
Perera et al. [22]	Household-based survey on knowledge, attitudes, and practices towards dengue infection and prevention in a semi-urban area (Ja-Ela MOH Area)	2021	Gampaha District	510	18+	Ja-Ela MOH Area	Good awareness; 56.5%, Good attitudes; 52.9%, Good practices; 50.7%
Radhika et al. [23]	Level of awareness of dengue disease among school children in Gampaha District, Sri Lanka, and effect of school-based health education programs on improving knowledge and practices	2019	Gampaha District	2194	13–15	School children	Knowledge prior to health education (HE) Good; 46.3% Excellent; 3% After HE 41.8% had excellent knowledge
Rajapaksha et al. [24]	health-seeking behaviors, dengue prevention behaviors and community capacity for sustainable dengue prevention in a highly dengue-endemic area, Sri Lanka	2023	Kurunegala District	496	18–70	Community in a high-endemic region	Preventive behaviors; 19.2% Early medical attention; 44.6%
Udaynga et al. [25]	Comprehensive evaluation of demographic, socio-economic and other associated risk factors affecting the occurrence of dengue incidence among Colombo and Kandy Districts of Sri Lanka: a cross-sectional study	2018	Colombo and Kandy	2000	0–55+	Community in high dengue incidence regions	dengue affected groups (test) had better knowledge on illness, than dengue-non-affected (control)
Udayanga et al. [26]	Socio-economic, knowledge, attitudes, and practices (KAP), household related and demographic based appearance of non-dengue infected individuals in high dengue risk areas of Kandy District, Sri Lanka	2018	Kandy District	1000	15–55+	Non-infected individuals in high-risk regions	Dengue knowledge; good-medium except in Kundasale and Kandy municipality Good preventive measures and high environmental cleanliness
Wickremasinghe et al. [27]	Knowledge, attitudes, and practices of a selected group of parents on pre-hospital management of fever in children in a dengue-endemic area of Southern Sri Lanka	2021	Teaching Hospital Karapitiya and District General Hospital, Matara	86	20–40+	Parents of children with fever hospitalized at Teaching Hospital Karapitiya and District General Hospital, Matara	Dengue risk awareness 27.9%, 55.8 knew NSAIDs were best avoided but 32.56 preferred NSAIDs for fever management 89.53% knew hydration was important

**Table 2:** Meta-analysis of the knowledge domain in dengue KAP surveys (2000–2023) in Sri Lanka.

Dengue Transmission Knowledge	No. Studies	Total Sample Size	Correct Responses	I^2	Random-Effects Model (95% CI)
Dengue is mosquito-borne	8	7255	6324	99.8%	87.3% [64–99.4%]
Vector species	7	5991	4322	99.4%	55.0% [34.7–74.5%]
Biting times of vector	7	5828	3647	98.5%	66.4% [52.8–78.8%]
Vector breeding characteristics	8	6128	5690	97.8%	88.8% [81.1–94.7%]
Disease knowledge					
Viral etiology	2	4384	3768	0%	86% [84.9–87%]
Serotypes	1	4000	2468	NA	61.7% [60.2–63.2%]
Febrile infection	8	6141	5514	99.7%	80.1% [53.1–97.2%]
Other dengue symptoms	2	462	98	98.9%	25.4% [0.3–70.%]
Recurrence of dengue	5	5144	3850	97.5%	0.68.4% [56.1–79.4%]
No specific medicine	3	419	180	97.4%	41.5% [15.4–70.4%]
Papaya leaf extract does not cure	2	282	113	78.6%	39.7% [27.7–52.3%]
Association with rainy season	4	1380	794	99.5%	65% [28.5–93.5%]

**Table 3:** Meta-analysis of the domain of attitudes in dengue KAP surveys (2000–2023) in Sri Lanka.

Illness Perceptions/Management	No. Studies	Total Sample Size	Correct Responses	I^2	Random-Effects Model (95% CI)
Dengue fatality	4	969	833	97.5%	83.1% [64.3–95.9%]
Preventable	1	200	152	NA	76.% [69.5–81.7%]
Susceptibility regardless of age	1	312	305	NA	97.8% [95.43–99.1%]
Treatable	2	812	758	93.7%	94.5% [86.4–99.2%]
Early treatment from a medical practitioner	7	2472	1929	98.4%	82.4% [68.7–92.9%]
Ayurveda as initial treatment	1	500	26	NA	5.2% [3.4–7.5%]
Hydration/oral fluids	5	1102	615	99%	56% [25.9–83.9%]
Control is the government’s responsibility	2	894	558	99.8%	73.6% [6.8–100.%]
Control is a self-responsibility	1	120	42	NA	35.% [26.5–44.2%]
Control is both individual and state responsibility	2	1010	584	96.3%	58.% [41.9–73.3%]
Support for premise inspection and/fogging	3	1647	1226	79.9%	75.3% [69.7–80.%]

**Table 4:** Meta-analysis of the practices domain in dengue KAP surveys (2000-2023) in Sri Lanka.

Preventive Practices	No. Studies	Total Sample	Correct Responses	I^2	Random-Effects Model (95% CI)
Scrubbing water containers	8	3312	2483	99.3%	72.4% [52.5–88.6%]
Remove water in outdoor receptacles	5	1234	1041	87.1%	86% [79.7–91.3%]
Remove water in indoor receptacles	3	1318	776	99.3%	58.3% [26.0–87%]
Roof-gutter management	2	996	444	95.3%	44.5% [30.7–58.8%]
Cover wells and tanks	2	537	478	0.0%	89.1% [86.3–91.6%]
Use mosquito nets	4	5100	2286	85.3%	45.7% [40.5–51%]
Protective clothing	1	137	75	NA	54.74 [46–63.3%]
Mosquito coils	3	4700	2930	99.4%	43.8% [16.9–72.8%]
Insecticides	3	1210	364	93.0%	27% [17.8–37.3%]
Repellant use	2	700	212	99.2%	19.3% [0- 63.2%]
Physical rest	2	696	492	94.6%	71.7% [56.3–84.9%]
Paracetamol as antipyretic	3	896	499	89.3%	57.3% [47.2–67.2%]
Avoid NSAIDs	2	849	269	96.8%	32.9% [16.6–51.7%]
Avoid dark colored drinks	3	981	267	99.5%	37.1% [2.6–82.8%]

## Data Availability

All relevant data generated and analyzed in the current study are included in the manuscript.

## Abbreviations

WHO	World Health Organization
DF	Dengue fever
DHF	Dengue haemrrhagic fever
DSS	Dengue shock syndrome
NDCU	National dengue control unit
IEC	Information, education, and communication
COMBI	Communication for behavioral impact
KAP	Knowledge, attitudes, and practices
MeSH	Medical subject headings
NSAID	Non-steroidal anti-inflammatory drug
